# The Effect of Airbnb Users’ Regret on Dissatisfaction and Negative Behavioral Intention

**DOI:** 10.3390/ijerph20010002

**Published:** 2022-12-20

**Authors:** Seunghwan Lee, Min Jung Kim, Dae-Young Kim

**Affiliations:** 1 Department of Tourism Management, Kongju National University, Gongju-si 32588, Choongnam, Republic of Korea; 2 Department of Hospitality Management, University of Missouri, Columbia, MO 65211, USA

**Keywords:** Airbnb, dissatisfaction, mental health, regret, stress, switching intention

## Abstract

As the most successful platform for peer-based accommodation sharing, Airbnb has transformed the lodging industry into something much more affordable and accessible for travelers on a budget. Compared to a hotel stay, however, its variability of facility and service has created guests’ negative emotions such as regret and dissatisfaction. These emotions may cause stress, which negatively affect mental health. Therefore, we explore the factors that influence Airbnb guests’ regret, and investigate the relationship between their regret, dissatisfaction, and negative behavioral intention. Structural equation model is utilized on a total of 456 U.S. consumers to examine the relationship among Airbnb users’ responses. The findings indicate that price perception influences regret and dissatisfaction the most. The study also reveals that regret has a positive correlation with dissatisfaction, while does not have an influence on switching intention and negative word of mouth. Based on the result, theoretical and managerial implications are discussed.

## 1. Introduction

Negative emotions may occur when consumers’ experience does not match their expectations. Many researchers have studied how the negative emotions (e.g., anger, anxiety, stress, and regret) impact consumers’ post-purchase decision making and behavioral responses (e.g., [1,2]). Of the negative emotions, regret has been examined particularly in the consumer behavior area, because it arises from comparing the performance of the chosen product or service with the performance of a forgone product or service. Regret is caused by a bad decision and is connected to self-blame [3]. It occurs when individuals recognize or imagine that their present situation would have been better had they acted differently. The consequences of regret are closely related to negative feedback and response [4,5] and could cause stress which negatively affect mental health [6]. To date, many medical experts have revealed psychological riskiness of regret. Fortz-Gray [6] reported that regret could ruin mental health resulting in chronic stress, negatively affecting hormonal and immune system functioning. Furthermore, repetitive regret may also result in predictive general stress [7]. This circumstance often happen in the process of post purchase behavior in the hospitality industry providing diverse options to decide. In this vein, it is critical to examine what brings about regret in this sector.

Applying the concept of sharing economy, Airbnb provides accommodation services through peer-to-peer networks [8]. This company offers alternatives to traditional accommodations by providing a personalized, localized, and sometimes more authentic experience to travelers [9]. However, recent articles have highlighted the negative aspects of Airbnb, such as privacy concerns [10,11], liability issues [12,13] and discrimination issues [14]. In terms of traveler experiences, recent media coverage and Airbnb community have reported Airbnb guests’ negative emotions that might cause regret. For example, drunken host intruded the room when the guest was sleeping, unprepared room without cleaning when they enter the room or host’s unilateral cancelling the room at the last minute [15]. In the early stage of Airbnb growth, the issues were acceptable for guests who sought for a budget accommodation or made their first-time transaction, because they could neither have high expectations nor service standards which cause disappointment. As Airbnb enters into luxury accommodations and the business travel market, however, one of the challenges is the maintenance of guests’ negative feelings and emotions toward the service variability. Moreover, considering these negative experiences are rarely happened at a hotel, regret is a meaningful aspect to explore Airbnb users’ experiences. Therefore, drawing from regret theory, this study examines how travelers behave after negative Airbnb experiences.

With the realizations, we argue that factors that create regret for Airbnb users include price perception, quality of facilities, social interactions, location, and online experience. First, researchers have asserted that economic benefit especially to young, and family travelers is considered to be a critical driver in the use of peer-to-peer accommodation companies (e.g., [16]). However, since most hosts utilize different strategies to set renting rates [17], unstable pricing might result in negative user emotions. Second, the quality and standards of room can vary considerably because Airbnb is not governed by set regulations. For this reason, users cannot be certain of the property’s condition before they book it. Third, Tussyadiah [18] asserted that interactions with hosts and local people are another reason why travelers choose Airbnb. However, Ambrosio [19] revealed many Airbnb users still experience no interactions or less interaction than their expectation with hosts or locals. Fourth, the location of Airbnb units has lower accessibility from public transportation hubs (e.g., subway stations, bus terminals, etc.) compared to that of hotel. In addition, guests can hardly find Airbnb units in some places because of uncertain information or their locations in crowded residential areas. Lastly, Tussyadiah and Pesonen [20] argue that unfamiliarity with the online system is another obstacle to using Airbnb.

Airbnb is a comparably new type of accommodation in the hospitality industry. Due to the rapid growth and success of this new company in this industry, many similar type of companies have launched such as HomeAway, Coachsurfing, Flipkey and Trampolinn. In the vacation rental industry, since 2010, Airbnb’s market share has increased intensely. According to statistical data, in 2022, Airbnb accounts for over 20% of the total peer to peer lodging market [21]. Even though many rental competitors try to go over Airbnb, this company is located in the first. Therefore, in this study we apply Airbnb which representing peer to peer accommodation industry and attempt to reveal how Airbnb users behave after they go through negative experiences. We examine Airbnb’s attributes and these effects on guests’ negative emotions, regret, dissatisfaction and negative behavioral intentions. The result of this study might contribute to the lodging industry after COVID-19 pandemic since this difficult situation allows travelers to stay at private and independent accommodation such as Airbnb [22]. To achieve the goal, this research presents following three purposes. First, we explore the factors related to Airbnb consumers’ negative experiences. Second, we examine the factors that influence their regret. Lastly, we investigate the relationship between consumers’ regret, dissatisfaction, and switching intention. This study will provide a better understanding and a more focused assessment on the dimensions of consumer needs and behavior associated with using Airbnb’s services. Examining Airbnb guests’ regret could provide meaningful suggestions how Airbnb manages their numerous hosts to go over traditional types of accommodations and helps Airbnb users to enjoy travel without stress. This current research also could improve regret theory in a hospitality marketing context by identifying Airbnb users’ behavior intention (i.e., switching intention) after they feel regret caused by negative experiences.

## 2. Literature Review

### 2.1. Regret

Regret is defined as a negative emotion predicated on an upward, self-focused, and counterfactual inference [3,23]. This phenomenon has emerged as a crucial feature of customer negative experiences. After the decision has been made, customers temporarily evaluate the alternatives as more similar than they actually are. In terms of regret, Bell [24] and Landman [25] proposed regret theory which is one of well-known alternatives to expected utility. They argued that a decision maker evaluating diverse prospects to choose considers not only by assessing the consequence of the selected prospect but also by pondering the alternative that the decision maker could have chosen. In this vein, if his or her choice did not exceed the expectation, the decision maker could perceive the regret which is negative emotion. In the marketing context, according to Tsiros [26], regret theory suggests that customers’ post-purchase behavior is composed of two constructs: satisfaction and regret. Specifically, the satisfaction construct implies the evaluation of the chosen prospect’s performance compared to customers’ pre-formed expectations, while the regret construct denotes a function of the selected prospect’s performance compared to the alternative’s outcome which could have been chosen. With regard to personal regret, numerous studies in diverse context have examined personal regret and related factors including (dis)satisfaction and customers’ post-consumption behavior (e.g., branding: [27]; Consumer behavior: [28]; Psychology: [3]).

In the context of tourism and hospitality, several scholars [4,5] have examined regret specifically. Jang et al. [4] attempt to examine the relationship among regret, disappointment, and negative behavioral intention (i.e., switching intention and negative word of mouth) in a restaurant setting. Their results indicate that both regret and disappointment are crucial determinants of customer dissatisfaction and negative behavioral intention. Specifically, regret is a key determinant of switching intention, whereas disappointment is a significant predictor of negative word of mouth. In addition, Mattila and Ro [5] also examine customers’ emotional responses when they face service failure in the restaurant sector. They conclude that customers who have experienced disappointment or regret tend to exhibit negative behaviors following dissatisfaction, such as negative word of mouth and switching.

In the context of Airbnb guest experiences, mass media have recently covered the negative issues [29,30]. For example, some guests receive a different room or house from what website described [29], or have hosts installing hidden cameras to watch guests and even sexual assaults taking place in Airbnb host rooms [30]. Under this circumstance, Airbnb guests’ negative experience enables them to think of its alternative, traditional hotel. Based on previous studies, this study attempts to examine Airbnb users’ negative experiences causing regret and dissatisfaction [27] and post purchase behavior such as switching intention [3].

### 2.2. Factors on Airbnb Users’ Negative Experiences

Many prior studies indicate that Airbnb differs significantly from traditional accommodations and have attempted to examine diverse factors that influence customer satisfaction [18,31] and customer attitudes [32]. In this current study, we extracted five factors—price perception, quality of facility, social interaction, location, and online experience—which influence customer negative experiences in selecting Airbnb. In regard to price perception, many scholars have presented economic benefit as one of the main drivers for customers when participating in the sharing economy [31,33]. They argue that price is generally considered as users’ self-benefit, which might be an important determinant of sharing systems. Customers’ perception of price also impacts their decision making and behavioral intention [34]. For example, customers generally evaluate the possible outcomes with alternatives of similar values through the price. Then, when they realize or perceive that some alternatives are better than others, they recognize an inequality [35]. Airbnb has been considered as less expensive alternative accommodation of traditional hotel, but statistical data suggests it is actually more expensive [36]. Hence, customers’ perceptions of price will lead to changes in their attitudes and buying behaviors [37].

Facility is seen as a unique characteristic of sharing accommodations that can influence customer negative experiences. Since all properties are individually owned, the quality of facility varies from room to room or house to house. Airbnb users are not able to assess this factor until they get into the room. Tussyadiah and Zach [38] argue that amenities and facilities at the residence are important attributes that guests use to evaluate of sharing accommodation. The third determinant in the framework refers to the social interaction involved with a sharing economy. Närvänen et al. [39] emphasize social interaction (e.g., community belonging) as a key role of sharing accommodation. In the context of Airbnb, Botsman and Rogers [33] assert that sharing activities contribute to satisfying consumers’ social needs, which include desire for socialization and sense of belonging [31]. Airbnb also announced that social interaction is one of the most differentiated factors compared to traditional hotels [40]. However, in real life, guests are commonly complaint about Airbnb because of a lack of or dissatisfactory interactions with other users or local hosts. Thus, it is reasonably assumed that social interaction will be a factor that influence Airbnb user negative experiences.

The fourth attribute that influences users’ post-purchase emotion is the location. In hotel industry, location plays a significant role in their successful operation [41] because ideal location is always related to greater accommodation demand [42], better performance [43], and lower failure rate [44]. However, Airbnb cannot consider location as hotel companies did because the majority of units are in residential areas where local people actually live. Thus, we assume that location-related issues on Airbnb (i.e., accessibility, lack of detailed information, etc.) might cause high levels of dissatisfaction. Fifth, online experience is the last factor influences users’ post-purchase emotion. All services Airbnb provides is driven by online environment [45]. This unique operation system might cause some occurrences that guests might feel negative. For example, company’s unilateral cancellation at the last minute or company’s allowance of evaluation of both host and guest each other that one of them might be disagree [46]. In this study, we examine dissatisfaction and regret with an Airbnb experience and its influence on post purchase behavior. Therefore, the first and second hypothesis are shown below:

**Hypothesis** **(H1):** *Airbnb users’ negative experiences will increase regret about their choice.*

H1a. Unrealistic perceptions of the price will increase regret about their choice.

H1b. Negative experiences regarding facilities will increase regret about their choice.

H1c. Negative experiences regarding social interactions will increase regret about their choice.

H1d. Negative experiences regarding about the location will increase regret about their choice.

H1e. Negative experiences regarding online experience will increase regret about their choice.

**Hypothesis** **(H2):** *Airbnb users’ negative experiences will increase their dissatisfaction.*

H2a. Unrealistic perceptions of the price will increase their dissatisfaction.

H2b. Negative experiences regarding facilities will increase dissatisfaction.

H2c. Negative experiences regarding social interactions will increase dissatisfaction.

H2d. Negative experiences regarding with the location will increase dissatisfaction.

H2e. Negative experiences regarding online experience will increase their dissatisfaction.

### 2.3. Regret, Dissatisfaction, and Negative Behavioral Intention

Regret influences customer’s responses and behaviors, such as purchase intentions and future purchase decisions [47,48]. As mentioned above, regret is caused by ‘bad decisions’, implying there would have been a better alternative. If that is the case, it is likely that customers will strive to choose the better alternative when they are faced with a similar situation in the future. When the customer considered all alternatives and the choice still turned out badly, it seems likely that switching would be a function of regret [49]. In this vein, many prior studies indicate a direct relationship between consumers’ negative emotions and their post-purchase behaviors (e.g., dissatisfaction, negative word of mouth and switching intention) [5,50,51] in the hospitality industry.

Scholars claim that dissatisfied consumers tend to switch companies more often than satisfied customers (e.g., [52,53]), which requires initiating a new relationship with another service provider. In addition, according to other findings by customer dissatisfaction results in negative word of mouth [54] and dissatisfied switchers tend to show higher post-switching negative word of mouth about the original company [55]. Hence, in this study, we presume a positive relationship between regret and negative post-purchase behavior (e.g., switching and negative word of mouth). Based on the literature, we propose the following hypotheses:

**Hypothesis** **(H3):** *Regret will increase users’ dissatisfaction.*

**Hypothesis** **(H4):** *Regret will increase users’ negative behavioral intentions.*

**Hypothesis** **(H5):** *Dissatisfaction will increase users’ negative behavioral intentions.*

**Figure 1:**
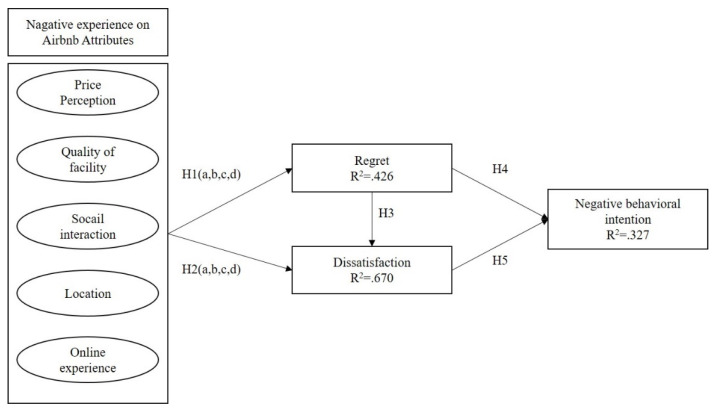
Research Model.

Based on Hypotheses proposed, this study also provided the research model (see Figure 1) as follow:

## 3. Methods

### 3.1. Sample and Data Collection

The data was collected through Amazon Mechanical Turk, an Internet consumer panel that connects researchers with a diverse group of consumers willing to participate in studies for modest monetary incentives. We focused on U.S. consumers who had used Airbnb within the last 12 months and possessed IP addresses in the United States. Many researchers have proven that Amazon MTurk online surveys produce reliable results that are consistent with other data collection sources [56]. We recruited 550 respondents to fill out surreys; however, 456 samples were used to analyze the data due to incomplete responses. Outliers were also removed, yielding a response rate of 83%.

### 3.2. Procedure and Measures

To ensure the appropriateness of the respondents, at the beginning of the online questionnaire, potential participants were asked to respond positively or negatively to the following statement: “I have had a negative experience regarding Airbnb within the last 12 months.” It means that the respondents should have Airbnb experience and they were not satisfied with their experiences. We generated rating scales to measure the antecedent factors. The first antecedent includes five items of price perception (e.g., “For the given quality of the Airbnb offer, I rated the price as bad”), and we asked how they perceived the price of the Airbnb unit. The second antecedent contains five items of quality of facility (e.g., “Mattress and pillow were uncomfortable”); participants reported how they felt about the quality of the Airbnb unit they used. The third antecedent includes seven items of social interactions (e.g., “Staying at Airbnb did not allow me to get insider tips on local attraction”), which described how much participants interacted with locals (i.e., attractions, neighbors, host, etc.). The fourth antecedent comprises four items concerning location (e.g., “It was not easy to access transportation from the Airbnb unit I stayed in.”), which addressed how respondents evaluated the Airbnb unit’s location. Finally, the fifth antecedents consist of three items related to the online experience (e.g., “They did not provide enough information about how it works”), which accounted for how they accessed their online experience, including information searches or reservation processes. These items of five factors in the survey were adapted from prior studies (e.g., [18,30,31,32,57]) and were slightly revised to fit into this study.

To measure Airbnb users’ level of regret after a negative experience, we adopted items from Creyer and Ross [58] such as “I knew that I should have chosen differently”, three of which measured dissatisfaction adopted from Zeithaml et al. [59] and Zeelenberg [60], such as “Overall, my negative experiences outweighed my positive experiences”, six of which measured negative behavioral intentions: three items of switching intention (i.e., “After the negative experience, I will not continue to use that Airbnb”), and three items of negative word of mouth (i.e., “I have discouraged friends and relatives from going to that Airbnb”) adopted from Zeelenberg [60]. The items are slightly changed to fit into this research (see Appendix A). In order to generate sufficient variance in responses, all items in this questionnaire were presented on a seven-point Likert-type scale, ranging from strongly disagree (1) to strongly agree (7) statements. Then, partial least squares structural equation modelling (PLS-SEM) was performed to test the hypotheses.

## 4. Results

### 4.1. Participants Information

The ages of the respondents ranged from 18 to over 65 years old, with approximately 40.6% in the 25 to 30 age range (*n* = 185). Fifty seven percent of the respondents were male (*n* = 260) and 43% were female (*n* = 196). The most common income range was $40,000 to $59,999, which was reported by 31.1% of the respondents (*n* = 142). Approximately 66% were Caucasian (*n* = 301). In terms of education level, almost half of the participants (45.2%) had baccalaureate (four year) degrees (*n* = 206). In order to decide whether or not to choose Airbnb, approximately 66.7% *(n* = 304) of the respondents compared it to a stay in a hotel. More than half (59.4%) of respondents (*n* = 271) had zero or one companion staying with them. Lastly, approximately, 79.2% (*n* = 361) of the respondents answered that their purpose for travel was leisure (See Table 1).

### 4.2. Data Analysis

To validate the measures developed and test the hypotheses, we conducted PLS-SEM using the Smart PLS 2.0 program [61]. By employing this method, we were able to effectively incorporate both reflective and formative measures and utilize fewer defensive assumptions about the data [62,63]. For example, because PLS uses bootstrapping to empirically estimate standard error to determine the parameter estimates, this method does not necessitate a normal distribution [64].

Scholars have differing opinions about the most effective sample size to use with this PLS model. For example, Chin [65] suggests that for each dependent variable, 20 cases would be most effective in this statistical model. Therefore, we took into consideration the number of structural paths and dependent variables. Specifically, Hair et al. [63] argue that it would be most effective to use ten times the largest number of structural paths directed at one particular construct in the inner path model. Thus, based on these recommendations, we chose 465 as the most valid sample size to achieve reliable results.

### 4.3. Measurement Model

Before examining the structural model, variance inflation factor (VIF) for the first-order factors of study variables was calculated to assess multicollinearity. As shown in Table 2, the VIF values are computed for three dimensions. Kock [66] indicated the cut-off value of a VIF is 3.3. According to his study, VIF value greater than 3.3, it is considered as an indication of pathological collinearity, and also as an indication that model may be contaminated by common method bias. However, in this study, since all VIFs resulting from a full collinearity test are lower than 3.3 (regret: 1.745; dissatisfaction: 3.030; negative behavioral intention: 1.486), there is no multicollinearity among the constructs and the study is considered free of common method bias.

Convergent validity and discriminant validity are determined through the assessment of reflective constructs, indicator reliability and internal consistency [67]. As shown in Table 3, the composite reliabilities range of 0.82 to 0.93 surpassed the recommended threshold value of 0.7 [16,68]. It should be noted that many researchers consider this value to be more appropriate for use with PLS-SEM than Cronbach’s alpha (e.g., [62]). To assess convergent validity, which is recommended by Fornell and Larcker [67,69], each construct’s AVE value was calculated (see Table 3).

Malhotra and dash [70] noted that “*AVE is a more conservative measure than CR. On the basis of CR alone, the researcher may conclude that the convergent validity of the construct is adequate, even though more than 50% of the variance is due to error*” (p. 702). Therefore, even though the AVE value for the facility (0.47) was a bit below 0.5, since AVE values for all other values were greater than the recommended level of 0.5 and all composite reliability values are over the suggested threshold value (0.7), convergent validity in this study is supported [69].

Hair et al. [62] stated that the cross-loadings are the typical approach to evaluate the discriminant validity of the indicators. As they suggested, we compared the indicator’s outer loadings and any of its cross-loadings (i.e., its correlation) on other constructs. To be specific, an indicator’s outer loadings on the related construct should be greater than any of its cross-loadings (i.e., its correlation) on other constructs. As shown in Table 4, all constructs’ outer loading (in bold) values are higher than any of its cross-loading values on other constructs. Therefore, based on the cross-loadings approach, in this study, discriminant validity has been established. In addition, to estimate the structural model, we utilized two types of assessments: the coefficient of determination (R2) and establishing the significant values of path coefficients [71]. Lastly, we estimated the predictive relevance of the model validity using Q2 values [64,72].

### 4.4. Hypothesis Testing

**Figure 2:**
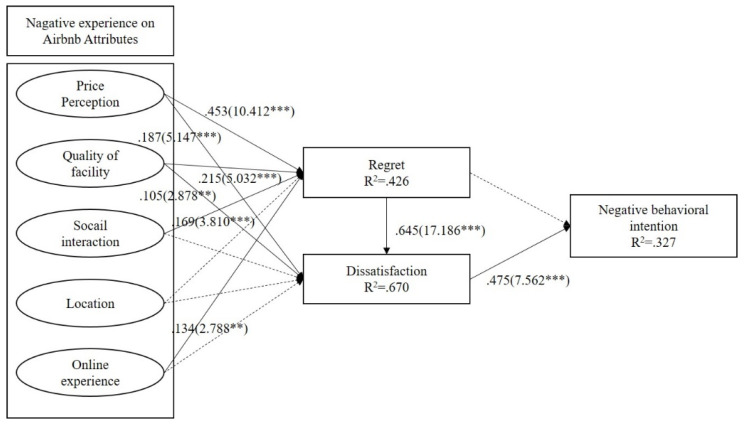
The Result of the Structural Model. Note. *** *p* < 0.001, ** *p* < 0.01.

In order to identify the determinants of regret, we estimated the structural model using Smart PLS and a bootstrap resampling method to obtain the *p*-values (see Figure 2). Table 5 illustrates the four significant determinants of regret that we identified: the positive effects of price perception (β = 0.453, *p* < 0.001), quality of facility (β = 0.215, *p* < 0.001), social interaction (β = 0.169, *p* > 0.001) and online experience (β = 0.134, *p* > 0.01). However, the impacts of location (β = 0.078, *p* = 1.895) on regret were not identified. Hence, our findings provided support for Hypotheses H1a, H1b, H1c and H1e, but did not support hypotheses H1d. For hypothesis 2, the results indicated that two dimensions of price perception (H2a) (β = 0.187, *p* < 0.001), a quality of facility (H2b) (β = 0.105, *p* < 0.001) were the factors that had a significant impact on dissatisfaction. Our tests revealed no significant effects of social interaction (H2c) (β = 0.016, *p* = 0.497), location (H2d) (β = −0.041, *p* = 1.201) and online experience (H2d) (β = 0.030, *p* = 0.778) on dissatisfaction. To test H3 through H5, we explored the relationships among regret, dissatisfaction and negative behavioral intention. As regret had a significant influence on dissatisfaction (H3) (β = 0.645, *p* < 0.001), but does not find significant impact on negative behavioral intention (H4) (β = 0.116, *p* = 1.875) and dissatisfaction had a significant influence on negative behavioral intention (H5) (β = 0.475, *p* < 0.001). The result supported H3 and H5, while H4 was not supported.

### 4.5. Mediating Effect of Regret and Dissatisfaction

The bootstrap method developed by Preacher and Hayes [73] is a non-parametric resampling test. The main feature of this test is that it does not rely on the assumption of normality [63]. This test has an advantage over Sobel’s test, and can help determine the mediation effect with certainty. In this approach, bootstrapping can be used twice: first without the presence of mediation, and secondly, with the presence of mediation. It should be noted that if the direct path is not significant, there is no mediating effect [74]. To examine mediating effect, we calculated the variance accounted for (VAF). VAF can be obtained by the value that (Indirect effect/Total effect).

According to Hair et al. [63], an outcome of VAF can be interpreted that if the VAF value is greater than 80%, full mediation is found, and if the VAF value is between 20% and 80%, it means partial mediation. The value less than 20% means there is no mediation. Through the mediating analysis, we found out the strength of mediation which is computed via VAF, as suggested by Hair et al. [63]. Table 6 reveals the effect of three dimensions of price perception (61.0%) and faciality (57.0%) on dissatisfaction is explained via regret. Since the all VAF values in the test presented between 20% and 80%, it can be said that regret partially mediates the relationship between two dimensions (i.e., price perception and facility) and dissatisfaction.

Lastly, to test the predictive power of the research model using PLS analysis, many scholars have turned to explained variance (R2) of the endogenous constructs [65] to determine the level of variance in the construct which is explained by the model. As shown in Figure 2, The R2 values of 0.426 (regret), 0.670 (dissatisfaction), and 0.327 (negative behavioral intention) indicate that the model has high predictive value and is capable of explaining endogenous constructs [75]. The predictive sample reuse technique has also been shown to have a high level of predictive relevance [65]. This technique, developed by Geisser [56] and Stone [72], is also known as the Stone-Geisser’s Q2. For this study, we calculated the Q2 values of the endogenous constructs in Smart PLS utilizing the blindfolding procedure. We found that all Q2 values are greater than ‘0’ (ranging from 0.211 to 0.513), which is indicative of the endogenous constructs’ high rate of predictive relevance (See Table 7).

**Figure 3:**
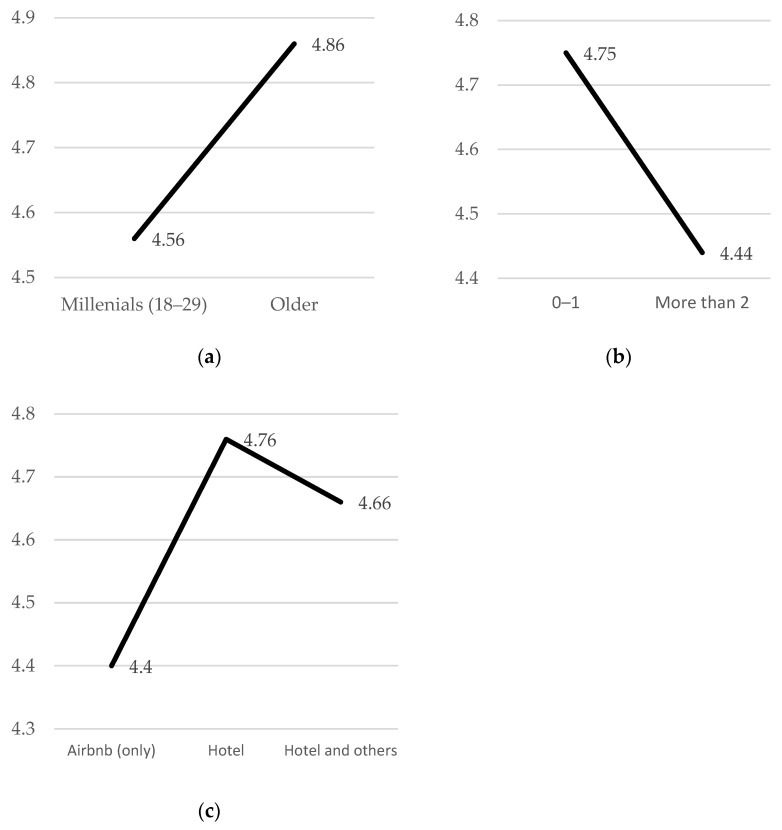
The result of ANOVA (Price perceptions and profiles). (**a**) price perception (y axis) and generation (x axis). (**b**) price perception (y axis) and visit numbers (x axis). (**c**) price perception (y axis) and accommodation type which they compared (x axis).

To clearly show how the results of this study would affect Airbnb users, we attempted to compare various groups of people (i.e., number of companions, age group, and types of compared accommodations) (See Table 8 and Figure 3). Since price perception was significantly associated with both regret and dissatisfaction, we focused on the price attribute. To analyze age group, we chose two groups: young millennials (18–29) and older (older than 29). We also further explored the effect of party size: one or two guests and the other group who with more than two companions. We have decided these numbers because maximum capacity without extra charge of general hotel room is two adults.

At last, regarding type of compared accommodations, we analyzed three groups: one group who chose only from Airbnb options, another group who compared Airbnb to hotels, and the other group who compared Airbnb facilities to both hotels and other accommodation companies. We found that age groups (F = 5.582, *p* < 0.05), number of companions (F = 7.586, *p* < 0.01), and accommodation groups (F = 3.112, *p* < 0.05) showed significant differences in terms of perceived price as an attribute of Airbnb. To be specific, millennials had fewer negative experiences than the older group. Our results also indicated that smaller groups had more negative experiences. Finally, those who compared Airbnb to a traditional hotel had more negative experiences than those who compared it to other Airbnb options.

## 5. Conclusions

### 5.1. Discussion

This study has attempted to analyze travelers’ emotions in Airbnb. To date, many related studies have focused on the positive side of this unique business venture. However, we examined the determinants of regret regarding utilizing Airbnb’s services and its effects on customers’ attitudes and post-purchase behaviors to extend the frontier of research on the peer-to-peer accommodation. We were able to identify the dominant aspects of Airbnb users’ experiences that cause their regret and dissatisfaction. This study assumed that Airbnb users’ negative emotions which derived from undesirable experience negatively influence on their mental health. As a result, this research found that four dimensions of price perception, facility, social interaction and online experience had significant effects on both users’ regret and two dimensions of price perception and quality of facility had significant influence on dissatisfaction.

We also confirmed positive relationships between regret and dissatisfaction and between dissatisfaction and negative behavioral intention, while regret did not have impact on negative behavioral intention. In addition, in order to apply the findings to real-world consumers, we attempted to figure out how various demographic groups responded to price perception which is the highest influence on regret and dissatisfaction. The results indicated that millennials, guests with larger parties, and groups who compared their accommodations only with other Airbnb options showed more satisfactory experiences regarding price perception than other groups in our comparisons. These findings show that diverse groups respond differently to Airbnb experiences, which will help marketers to identify successful potential service strategies and understand the unique characteristics of potential consumers.

In this study, it is very critical to say that the results of cleanliness and quality of facility are closely related to the corona pandemic that the current society is going through. The COVID-19 has a significant influence on the travel industry. It would be very important to consider how travelers use Airbnb and perceive this organization during this corona pandemic. In this specific circumstance, while travelers are staying with Airbnb, cleanliness and quality of facilities in each units of Airbnb have been very critical factors. Even after this pandemic, the importance of these factors will continue to increase. However, paradoxically, the importance of social interaction could decrease after this unexpected situation. To date, Airbnb has concentrated on the diverse interaction with the local people, COVID-19 would be able to change individuals’ thoughts regarding social interaction on tourism destination.

### 5.2. Theoretical Implication

The results of this study suggest meaningful theoretical implications for researchers. This research contributes to (1) understanding regret in the context of Airbnb, (2) indicating five different factors which influence regret and dissatisfaction in the Airbnb context, and (3) advancing comprehension of the relationship among regret, dissatisfaction and post behavioral intention in the context of Airbnb. First, this study expanded the measurement of negative emotions in the context of Airbnb. To date, some studies have examined regret and dissatisfaction, but the background of studies was the restaurant industry. [4,76]. In this study, we could reveal characteristics of guests’ emotion in sharing accommodation sector in service industry. In addition, many prior studies have attempted to show several determinants to fulfil customers’ satisfaction when they use a peer-to-peer accommodation [18,31]. The unique contribution of this study is to shed a light on Airbnb guests’ negative emotion. The role of guests’ negative emotion could provide more meaningful consequences to understand guests in service industry. The results can be used as a basis for future Airbnb brand studies and comparison studies with hotel brands.

Second, this study assumed that five different factors adapted from prior studies might influence on regret and dissatisfaction of Airbnb guests. However, this study proved that regret and dissatisfaction are predicted by discrete factors. To be specific, the result of this study was able to present four factors which could affect guests’ regret (i.e., price perception, facility, social interaction, and online experience) and two factors which could affect dissatisfaction (i.e., price perception, facility). Surprisingly, we were not able to find any statistical support for determinants primarily discussed in more recent research contributions, stressing the critical role of location. The consequence of this study might be able to support future research to unveil the role of other negative emotions such as anger, frustration or guilt.

Third, this study revealed and established the relationship between regret, dissatisfaction and post behavioral intention in the context of sharing economy. Inconsistent with our expectations, the results indicated that regret was not a significant determinant of negative behavioral intention but a significant predictor of dissatisfaction which has a positive impact on negative behavioral intention. This result is incompatible with prior studies [3,4,27] which showed positive relationship between regret and negative behavioral intention. This study findings have enriched the understanding of how the particular negative emotion affects guests’ dissatisfaction and post behavioral intention in the context of sharing economy, where was recently emerged.

### 5.3. Managerial Implication

The findings of this study also offer several important managerial implications to Airbnb operators and marketers. First, the result stated that price perception and quality of facility showed direct effects on both regret and dissatisfaction revealing their importance for Airbnb users. Hence, marketers should consider establishing basic standards and guidelines to prevent their regret and decrease their dissatisfaction. Based on this result, we suggest that the company focus more on educating the hosts regarding the significance of effective pricing strategies. In addition, quality of facility is also important variable to cause negative emotion because unidentical facilities in each unit could make users hesitate to clearly select to stay. Therefore, marketers also need to consider determining minimum required facilities that should be equipped in each unit. This might help guests maintain their staying without basic complaints. Then, Airbnb marketers also should be able to consider how to standardize the quality of facility in each Airbnb unit. It would be a good idea to make a basic standard manual including facilities every Airbnb unit should be provided.

Second, since we found the importance of price perception, this study examined price perception connecting with demographic information such as age group, number of companions, and types of different accommodations that guests have compared before making a decision. The result could provide more notable and effective implication. It shows that millennials were less dissatisfied with Airbnb rates than older groups. As many studies mentioned, millennials are the majority users of Airbnb. However, it does not mean that their marketing strategy focus only on young travelers. Even though it is very true that the old groups prefer traditional accommodation such as hotel, to broad their market and improve their business, they should consider silver age travelers as their potential guests. For example, with acceptable price of Airbnb unit, firm is able to attract them by providing senior community to interact with similar age groups in destination.

In addition, the result that examined the number of companions revealed that guests with fewer companions (zero or one) were less satisfied with Airbnb rates than guests with more companions. Most of hosts set a specific maximum capacity per unit and that number is normally greater than the general hotel capacities for one room. Therefore, travelers can assume that Airbnb can be an ideal accommodation for group travelers. For this reason, guests with fewer companions or solo travelers might consider the rates as comparably expensive, which can cause switching accommodation. This result can suggest that Airbnb needs to focus on solo travelers providing discount promotions to groups belonging fewer people. According to Airbnb, in fact, recently, after COVID-19, solo travelers are more likely to reserve Airbnb than traditional accommodation. In 2022, 50 percent of all users booked for long term stay were solo travelers, while 26 percent of all booked in 2021 [40]. The comparison study finally showed that travelers were not satisfied with the price when different types of accommodations (e.g., hotels or resort) were compared with Airbnb. In terms of price of this company, the most important issue is users’ perceived price sensitivity. Likewise, this company needs to perform diverse price strategies to overcome users’ price sensitivity.

Third, one of interesting results is a significant effect of social interaction on regret. Social interaction is unique characteristic that guests can experience only through Airbnb, but guests can feel regret on social interaction, and it does not have significant effect on switching intention. This suggests that the guests could find another unit on Airbnb to experience social interaction. Airbnb marketers should consider social interaction more seriously. Based on the result, we argue that guests want to confirm the availability of interaction with local people including host. Many Airbnb guests expect to interact with local residents as the company’s basic slogan, ‘live like a local’. However, since every host has different condition and schedule, guests are not able to be guaranteed by host to experience interaction. Therefore, it would be a good idea for hosts to inform what they can provide guests such as offering useful local information or having a meal together on their web site. Then, it would be very helpful for guests who want to interact with hosts while their staying.

Lastly, in terms of the relationship among regret, dissatisfaction, and negative behavioral intention, it can be interpreted based on one recent study conducted by Davidai and Gilovich [77]. People generally feel regret when they find better alternative than what they already choose. It means that regret is posed by what people could have done, not by what people did do wrong or the service was too bad. Therefore, unless they are satisfied with the products or service of Airbnb, they might switch to different lodging types, otherwise, they may be able to continue to decide Airbnb as their accommodation for their next trip. Based on their argument, in this context, Airbnb professionals or hosts should let guests feel that choosing Airbnb rather than traditional hotels was not a bad decision. Therefore, Airbnb marketers should figure out what exact features their guests desire to experience compared to other competitors and attempt to fulfill their satisfaction.

### 5.4. Limitation

We have identified some limitations in this study, suggesting that further research should be conducted. We used a convenience sample, and its data were collected from only one country, US. We, therefore, cannot be widely generalized. Future studies may want to use a probability sampling method and data collected from multiple countries to increase external validity. Since Airbnb has been operated in a different way compared to traditional accommodations such as hotels or resorts, Airbnb experience cannot fully represent the experience across different accommodation segments. Hence, conclusions should be drawn only with caution. In addition, over time, many new lodging companies and alternative types of accommodations have emerged. For example, with Airbnb’s increasing popularity, many online travel agencies (e.g., Expedia and Priceline) began providing vacation rentals. In future studies, by analyzing and comparing various accommodation types, including traditional hotels, Airbnb and its continually emerging competitors could yield interesting and insightful results. Finally, as aforementioned in the section of discussion, the corona pandemic would be able to alter the results of this study. Therefore, for the future studies, it will be interesting and meaningful to discover the different factors that influence on personal regret before and after the corona pandemic.

## Figures and Tables

**Table 1:** Characteristics of respondents (*n* = 456).

	#	%		#	%
Gender			Travel purpose		
Male	260	57.0	Leisure	361	79.2
Female	196	43.0	Business	81	17.8
			Other	14	3.1
Age					
18–24	80	17.5	Information Source (what information they use for decision making)		
25–30	185	40.6	Online search	326	71.5
31–40	134	29.4	Banner	12	2.6
41–50	31	6.8	News Paper, TV or Radio Ads	17	3.7
51–60	19	4.2	Friends	96	21.1
Over 61	7	1.5	Others	5	1.1
Education Level			Comparison against (when they decide Airbnb)		
High school or less	35	7.7	None (only Airbnb)	85	18.6
Some college or associate (2 year) degree	149	32.7	Hotels	304	66.7
Baccalaureate (4 year) degree	206	45.2	Other accommodations	16	3.5
Graduate studies/post-graduate studies	66	14.5	Hotels and other accommodations	51	11.2
Ethnicity			# of companions		
White/Caucasian	301	66.0	Alone (no companion)	72	15.8
African American	59	12.9	1 companion	199	43.6
Hispanic/Latino American	30	6.6	2 companions	87	19.1
Asian	46	10.1	3 companions	41	9.0
American Indian/Native American	7	1.5	4 companions	21	4.6
Other Ethnic Background	7	1.5	5 companions	18	3.9
Prefer not to answer	6	1.3	More than 5 companions	18	3.9
Annual Household Income					
$0–20,000	62	13.6			
$20,000–39,999	71	15.6			
$40,000–59,999	142	31.1			
$60,000–79,999	70	15.4			
$80,000–99,999	55	12.1			
$100,000+	47	10.3			

**Table 2:** Result of variance inflation factor (VIF).

	R2	1-R2	VIF
Regret	0.427	0.573	1.745
Dissatisfaction	0.670	0.330	3.030
Negative Behavioral Intention	0.327	0.673	1.486

**Table 3:** Latent variable correlation.

	PP	FA	SI	LO	OE	RE	DS	NBI	Composite Reliability
PP	**0.64**								**0.90**
FA	0.43	**0.47**							**0.82**
SI	0.28	0.30	**0.60**						**0.91**
LO	0.16	0.24	0.36	**0.77**					**0.93**
OE	−0.18	−0.32	−0.39	−0.38	**0.73**				**0.85**
RE	0.59	0.44	0.35	0.22	−0.16	**0.64**			**0.90**
DS	0.60	0.46	0.30	0.15	−0.10	0.80	**0.78**		**0.92**
NBI	0.41	0.38	0.31	0.19	−0.32	0.50	0.57	**0.68**	**0.93**

Note: DS-dissatisfaction, FA-quality of facility, LO-location, NBI-negative behavior intention, OE-online experience, RE-regret, SI-social interaction. Note: Reliability is calculated by internal consistency reliability; Items on the diagonal (in bold) represent AVE scores.

**Table 4:** PLS confirmatory factor analysis for discriminant and convergent validity.

	Price Perception	Facility	Social Interaction	Location	Online Experience	Regret	Dissatisfaction	Negative Behavioral Intention
**P1**	**0.76**	**0.33**	**0.20**	**0.08**	**−0.14**	**0.43**	**0.47**	**0.36**
**P2**	**0.79**	**0.36**	**0.21**	**0.13**	**−0.11**	**0.47**	**0.46**	**0.27**
**P3**	**0.82**	**0.34**	**0.23**	**0.16**	**−0.15**	**0.48**	**0.47**	**0.33**
**P4**	**0.81**	**0.36**	**0.26**	**0.09**	**−0.12**	**0.49**	**0.54**	**0.35**
**P5**	**0.82**	**0.34**	**0.23**	**0.19**	**−0.19**	**0.48**	**0.46**	**0.34**
**FA1**	**0.32**	**0.75**	**0.15**	**0.13**	**−0.24**	**0.32**	**0.34**	**0.26**
**FA2**	**0.32**	**0.70**	**0.16**	**0.21**	**−0.26**	**0.31**	**0.33**	**0.29**
**FA3**	**0.19**	**0.62**	**0.24**	**0.17**	**−0.23**	**0.25**	**0.25**	**0.28**
**FA4**	**0.32**	**0.73**	**0.25**	**0.10**	**−0.16**	**0.32**	**0.33**	**0.23**
**FA5**	**0.31**	**0.61**	**0.24**	**0.21**	**−0.20**	**0.30**	**0.31**	**0.23**
**SI1**	**0.24**	**0.25**	**0.71**	**0.31**	**−0.32**	**0.27**	**0.23**	**0.24**
**SI2**	**0.23**	**0.23**	**0.79**	**0.25**	**−0.32**	**0.27**	**0.26**	**0.24**
**SI3**	**0.17**	**0.21**	**0.79**	**0.36**	**−0.36**	**0.28**	**0.20**	**0.25**
**SI4**	**0.22**	**0.19**	**0.79**	**0.31**	**−0.30**	**0.29**	**0.22**	**0.26**
**SI5**	**0.24**	**0.24**	**0.80**	**0.24**	**−0.24**	**0.28**	**0.28**	**0.20**
**SI6**	**0.25**	**0.28**	**0.73**	**0.32**	**−0.35**	**0.24**	**0.21**	**0.24**
**SI7**	**0.18**	**0.21**	**0.76**	**0.19**	**−0.23**	**0.25**	**0.21**	**0.24**
**LO1**	**0.15**	**0.22**	**0.28**	**0.82**	**−0.35**	**0.15**	**0.12**	**0.12**
**LO2**	**0.11**	**0.17**	**0.27**	**0.89**	**−0.32**	**0.20**	**0.13**	**0.15**
**LO3**	**0.16**	**0.22**	**0.35**	**0.89**	**−0.33**	**0.20**	**0.13**	**0.19**
**LO4**	**0.15**	**0.23**	**0.37**	**0.90**	**−0.35**	**0.22**	**0.16**	**0.18**
**OE1**	**−0.15**	**−0.25**	**−0.35**	**−0.32**	**0.90**	**−0.18**	**−0.09**	**−0.21**
**OE2**	**−0.12**	**−0.23**	**−0.24**	**−0.34**	**0.70**	**−0.06**	**−0.03**	**−0.31**
**OE3**	**−0.13**	**−0.27**	**−0.28**	**−0.23**	**0.62**	**−0.06**	**−0.07**	**−0.32**
**RE1**	**0.51**	**0.40**	**0.23**	**0.14**	**−0.10**	**0.84**	**0.70**	**0.37**
**RE2**	**0.47**	**0.36**	**0.31**	**0.21**	**−0.18**	**0.84**	**0.64**	**0.39**
**RE5**	**0.50**	**0.34**	**0.26**	**0.16**	**−0.05**	**0.80**	**0.61**	**0.30**
**RE6**	**0.45**	**0.30**	**0.26**	**0.20**	**−0.04**	**0.79**	**0.60**	**0.33**
**RE8**	**0.42**	**0.36**	**0.33**	**0.19**	**−0.26**	**0.73**	**0.62**	**0.57**
**DS1**	**0.52**	**0.39**	**0.26**	**0.12**	**−0.09**	**0.70**	**0.89**	**0.49**
**DS2**	**0.53**	**0.40**	**0.28**	**0.13**	**−0.11**	**0.67**	**0.87**	**0.52**
**DS3**	**0.55**	**0.43**	**0.26**	**0.15**	**−0.05**	**0.73**	**0.90**	**0.50**
**NBI1**	**0.34**	**0.28**	**0.25**	**0.08**	**−0.19**	**0.44**	**0.51**	**0.78**
**NBI2**	**0.36**	**0.29**	**0.28**	**0.20**	**−0.30**	**0.39**	**0.47**	**0.86**
**NBI3**	**0.32**	**0.26**	**0.24**	**0.18**	**−0.27**	**0.34**	**0.45**	**0.83**
**NBI4**	**0.34**	**0.36**	**0.21**	**0.12**	**−0.23**	**0.46**	**0.50**	**0.77**
**NBI5**	**0.35**	**0.31**	**0.25**	**0.15**	**−0.30**	**0.41**	**0.45**	**0.85**
**NBI6**	**0.31**	**0.35**	**0.29**	**0.20**	**−0.32**	**0.38**	**0.42**	**0.85**

Note: In this table, rows for the indicators and columns for the latent variables. Note: Values in bold represent outer loadings on the associated constructs.

**Table 5:** Structural parameter estimates.

	Path	Path Coefficient	*t*-Value	Result
H1-1	Price perception → Regret	0.453	10.412 ***	supported
H1-2	Quality of facility → Regret	0.215	5.032 ***	Supported
H1-3	Social interaction → Regret	0.169	3.810 ***	Supported
H1-4	Location → Regret	0.078	1.895	Not supported
H1-5	Online experience → Regret	0.134	2.788 **	Supported
H2-1	Price perception → Dissatisfaction	0.187	5.147 ***	Supported
H2-2	Quality of facility → Dissatisfaction	0.105	2.878 **	Supported
H2-3	Social interaction → Dissatisfaction	0.016	0.497	Not supported
H2-4	Location → Dissatisfaction	−0.041	1.201	Not supported
H2-5	Online experience → Dissatisfaction	0.030	0.778	Not supported
H3	Regret → Dissatisfaction	0.645	17.186 ***	Supported
H4	Regret → Negative behavioral intention	0.116	1.875	Not supported
H5	Dissatisfaction → Negative behavioral intention	0.475	7.562 ***	Supported

Note. *** *p* < 0.001, ** *p* < 0.01.

**Table 6:** Result of the VAF test for the mediating effect of regret.

Path	Direct Effect	Indirect Effect	Total Effect	Variance Account for (VAF)
Price perception → Regret	0.453	-	0.453	-
Price perception → Dissatisfaction	0.187	0.292	0.479	0.610
Regret → Dissatisfaction	0.645	-	0.645	-
Quality of facility → Regret	0.215	-	0.215	-
Quality of facility → Dissatisfaction	0.105	0.139	0.244	0.570
Regret → Dissatisfaction	0.645	-	0.645	-

Note: Variance account for (VAF) = Indirect effect/Total effect.

**Table 7:** The results of predictive relevance.

	SSO	SSE	Q^2^ (1-SSE/SSO)
Regret	2520	1875.121	0.256
Dissatisfaction	1512	736.723	0.513
Negative behavioral intention	3024	2386.242	0.211

Note: SSO refers to Sum of squares of observations for one manifest variable; SSE refers to Sum of squared prediction errors for one manifest variable.

**Table 8:** The result of ANOVA.

		Sum of Squares	df	Mean Square	F	Sig.
Age group (Young Millennials vs. Older)	Between groups	7.726	1	7.726	5.582	**0.019**
	Within groups	628.418	454	1.384		
	Total	638.144	455			
Companion (0–1 vs. above 2)	Between groups	10.454	1	10.454	7.586	**0.006**
	Within groups	625.690	454	1.378		
	Total	636.144	455			
Compared accommodations (Airbnb (only) vs. Hotel vs. Hotel and others)	Between groups	8.512	2	4.256	3.112	**0.045**
	Within groups	616.872	451	1.368		
	Total	625.382	453			

## Data Availability

All datasets are available from the corresponding authors by reasonable request.

## Appendix A

### Appendix A.1. Negative Experience on Airbnb Attributes (5 Attributes: Price Perception, Quality of Facility, Social Interaction, Location, and Online Experience)

* reverse coded items.

#### Appendix A.1.1. Price Perception

P1. For the given price, I rated the Airbnb offer as bad

P2. For the given quality of the Airbnb offer, I rated the price as bad

P3. The price of the Airbnb was appropriate relative to its performance *

P4. The price of the Airbnb did not meet my expectations

P5. The price of the Airbnb was acceptable *

#### Appendix A.1.2. Quality of Facility

F1. The room was not clean

F2. The room was ready as promised *

F3. Mattress and pillow were uncomfortable

F4. Bathroom and shower facilities were bad

F5. All facilities were not provided as the host was supposed to

#### Appendix A.1.3. Social Interaction

S1. Staying at Airbnb did not allow me to get insider tips on local attractions

S2. Staying at Airbnb did allow me to have a more meaningful interaction with locals *

S3. It was difficult getting to know people from the local neighborhoods

S4. It was difficult developing social relationships

S5. Staying at Airbnb helped me connect with locals *

S6. It was difficult being part of a group of like-minded people

S7. The use of Airbnb did not allow me to belong to a group of people with similar interests

#### Appendix A.1.4. Location

L1. It was not easy to access transportation from the Airbnb unit I stayed in

L2. It was not easy to find a restaurant near the Airbnb unit I stayed in

L3. The Airbnb unit I stayed was close to shopping *

L4. The Airbnb unit I stayed was far to travel attractions

#### Appendix A.1.5. Online Experience

O1. They did not provide enough information about how it works

O2. It was difficult to search for the list of vacation rentals online

O3. I do trust online platform to execute the transaction *

### Appendix A.2. Regret

R1. I regret my choice

R2. I think I made an error in judgment

R3. Before I received outcome feedback, I knew that I had made an excellent decision

R4. I am confident I made the best choice based on the information I had available

R5. Before I should have chosen differently

R6. I knew that I should have chosen differently

R7. I really feel good about my choice

R8. I really feel that I was making an error when I made that choice

### Appendix A.3. Dissatisfaction

D1. On the whole, I was dissatisfied with my experience with the services

D2. Overall, my negative experiences outweighed my positive experiences

D3. In general, I was unhappy with staying at Airbnb

### Appendix A.4. Switching Intention

SI1. After the negative experience, I have not continued to use Airbnb

SI2. I will probably not use the services of that Airbnb in the future

SI3. I will definitely not return to that Airbnb in the future

SI4. I have said negative things about the Airbnb to other people

SI5. I have discouraged friends and relatives from going to that Airbnb

SI6. I have advised against the Airbnb when someone sought my advice
